# Impact of guidelines implementation on empiric antibiotic treatment for pediatric uncomplicated osteomyelitis and septic arthritis over a ten-year period: Results of the ELECTRIC study (ostEomyeLitis and sEptiC arThritis tReatment in children)

**DOI:** 10.3389/fped.2023.1135319

**Published:** 2023-02-23

**Authors:** Chiara Minotti, Francesca Tirelli, Chiara Guariento, Giulia Sturniolo, Carlo Giaquinto, Liviana Da Dalt, Francesco Zulian, Alessandra Meneghel, Giorgia Martini, Daniele Donà

**Affiliations:** ^1^ Division of Pediatric Infectious Diseases, Department of Women’s and Children’s Health, University of Padua, Padua, Italy; ^2^ Pediatric Rheumatology Unit, Department of Women’s and Children’s Health, University of Padua, Padua, Italy; ^3^ Pediatric Emergency Department, Department of Women’s and Children’s Health, University of Padua, Padua, Italy

**Keywords:** children, bone and joint, infection, osteomyelitis, septic arthritis, antimicrobial stewardship (AMS), narrow spectrum, empiric treatment

## Abstract

**Background:**

Due to the growing evidence of the efficacy of intravenous (IV) cefazolin with an early switch to oral cefalexin in uncomplicated pediatric osteomyelitis (OM) and septic arthritis (SA) in children, we changed our guidelines for empiric antibiotic therapy in these conditions. This study aims at evaluating the impact of the guidelines' implementation in reducing broad-spectrum antibiotic prescriptions, duration of IV antibiotic treatment and hospital stay, treatment failure and recurrence.

**Materials and methods:**

This is a retrospective, observational, quasi-experimental study. The four years pre-intervention were compared to the six years, ten months post-intervention (January 2012, through December 2015; January 2016, through October 31st, 2022). All patients aged 3 months to 18 years with OM or SA were evaluated for inclusion. Each population was divided into three groups: pre-intervention, post-intervention not following the guidelines, and post-intervention following the guidelines. Differences in antibiotic prescriptions such as Days of Therapy (DOT), activity spectrum and Length of Therapy (LOT), length of hospital stay (LOS), broad-spectrum antibiotics duration (bsDOT), treatment failure and relapse at six months were analyzed as outcomes.

**Results:**

Of 87 included patients, 48 were diagnosed with OM (8 pre-intervention, 9 post-intervention not following the guidelines and 31 post-intervention following the guidelines) and 39 with SA (9 pre-intervention, 12 post-intervention not following the guidelines and 18 post-intervention following the guidelines). In OM patients, IV DOT, DOT/LOT ratio, and bsDOT were significantly lower in the guidelines group, with also the lowest proportion of patients discharged on IV treatment. Notably, significantly fewer cases required surgery in the post-intervention groups. Considering SA, LOS, IV DOT, DOT/LOT ratio, and bsDOT were significantly lower in the guidelines group. The treatment failure rate was comparable among all groups for both OM and SA. There were no relapse cases. The overall adherence was between 72 and 100%.

**Conclusions:**

The implementation of guidelines was effective in decreasing the extensive use of broad-spectrum antibiotics and combination therapy for both OM and SA. Our results show the applicability, safety, and efficacy of a narrow-spectrum IV empirical antibiotic regimen with cefazolin, followed by oral monotherapy with first/second-generation cephalosporins, which was non-inferior to broad-spectrum regimens.

## Introduction

Acute bacterial osteomyelitis (OM) and septic arthritis (SA) occur more commonly in pediatric age than in adults, particularly in children younger than five years of age (1, 2). Incidence of OM and SA is estimated to be 1.2 to 13 cases per 100,000 children per year and 1 to 37 cases per 100,000, respectively, depending on the setting and population (1, 3–6). Considering all age categories, *Staphylococcus aureus* is the most commonly responsible pathogen, while *Kingella kingae* is especially widespread in younger patients (<3 years). The empiric therapeutic approach is heterogeneous in Europe, as reported by several surveys and by the most recent guidelines for bone and joint infections (BJI) by the European Society for Pediatric Infectious Diseases (ESPID) (5, 7). The most appropriate options for oral antibiotic treatment in BJI are still under discussion, together with the optimal timing for the shift from intravenous (IV) to oral therapy. Empiric treatment should start promptly, possibly after sample collection for microbiological analysis. It should provide optimal coverage for methicillin-sensitive *S. aureus* (MSSA) and *K. kingae*, and for methicillin-resistant *S. aureus* (MRSA) in areas where its prevalence is higher than 10%–15% (7). Anti-staphylococcal penicillins and first-generation cephalosporins are the antibiotics of choice in contexts with a low MRSA prevalence. Clindamycin has also been reported for BJI treatment, in case of elevated MRSA prevalence (5–7). In case of uncomplicated infections, an early shift to oral antibiotics is possible and suitable in most patients, with a favorable outcome, as long as a total treatment duration of at least three-four weeks for OM and two-three weeks for SA is ensured (7). Follow-up treatment should be tailored on the susceptibility patterns of the antibiogram of the responsible pathogens, if isolated. *Salmonella* spp, MRSA or Panton-Valentine leukocidin (PVL) positive staphylococcal strains are responsible for complicated infections. Those cases, as well as BJI in young patients and those showing a slow clinical improvement, may benefit from a longer IV and oral antibiotic course. Factors associated with more severe sequelae are infection by invasive strains, a late start of therapy after symptoms onset and hip involvement. Patients presenting such features require a close and sometimes prolonged follow-up (7).

This study aims to assess the impact of the implementation of guidelines, suggesting the use of IV first generation cephalosporins (cefazolin) with an early switch to oral first/second generation cephalosporins (cefalexin/cefuroxime axetil) for the empiric treatment of uncomplicated OM/SA in children aged 3 months to 18 years.

## Materials and methods

### Study design and intervention

This is a monocentric, retrospective, observational, pre-post, quasi-experimental study, set at the Department of Women's and Children's health in Padua, Northern Italy.

Between the end of 2015 and the beginning of 2016, OM/SA internal guidelines were developed by the Division of Pediatric Infectious Diseases and the Pediatric Rheumatology Unit of Padua University Hospital, summarizing international literature evidence. In addition, three training sessions with an overview of the guidelines and treatment rationale were offered to attending physicians and residents.

The impact of the intervention was assessed by comparing the four-year period before OM/SA guidelines implementation (pre-intervention: January 1st, 2012, through December 31st, 2015) to the six years and ten months after intervention (post-intervention: January 1st, 2016, through October 31st, 2022).

According to the implemented guidelines, in fully vaccinated patients older than 3 months, an IV empirical antibiotic therapy is started with a first-generation cephalosporin (cefazolin 150–200 mg/kg/day) for 5–7 days in uncomplicated forms, as the prevalence of MSSA is above 90% in the considered area (7, 8). The subsequent shift in case of identification of the causative microorganism is to targeted oral therapy, otherwise to an oral antibiotic with the same spectrum activity as the IV therapy (shift from cefazolin to cefalexin or cefuroxime axetil). The total suggested duration of OM treatment is three-four weeks in case of clinical improvement with a normalized C-reactive protein (CRP) before the twentieth day of therapy. The total duration of SA is two-three weeks if isolated, or four weeks in case of associated OM (7).

Broad-spectrum antimicrobials were defined as: β-lactam and β-lactamase inhibitor combinations, third-generation cephalosporins, clindamycin, glycopeptides, fluoroquinolones, and macrolides. Therapeutic regimens including at least one broad-spectrum prescription, despite the association with amoxicillin or oxacillin, were considered broad-spectrum.

### Population, case definition, inclusion and exclusion criteria

All consecutive patients aged 3 months to 18 years old with a diagnosis of acute OM and/or SA according to the International Classification of Diseases, 9th Revision, Clinical Modification code were evaluated for inclusion. A case was defined by diagnosis of OM or SA on imaging, preferably magnetic resonance imaging (MRI, gold standard) for OM, or in alternative computed tomography (CT scan), Tc99 bone scintiscan, PET-TC scan, or ultrasound (US)/MRI for SA. Long bones were considered the typical site of infection for OM. The hips were considered a high-risk site for both OM and SA. Exclusion criteria were diagnosis of immunodeficiency or hemoglobinopathy or chronic granulomatous disease, immunosuppressive therapy, concomitant systemic bacterial infection, and ongoing antibiotic treatment on admission. Patients with complicated infections, not fully vaccinated, and/or with incomplete follow-up were excluded, as well as those with chronic osteomyelitis and Brodie's abscess.

The population was divided into two main groups, OM and SA. Each group was further divided into three groups: pre-intervention, post-intervention not following the guidelines (no GL), and post-intervention group with adherence to the guidelines (GL).

The following variables, selected *a priori*, were evaluated: age, sex, weight, fever, vaccination status, white blood cells, and neutrophil count, CRP, erythrocyte sedimentation rate (ESR), and procalcitonin (PCT) at onset, IV and oral antibiotic treatment with duration, diagnosis and imaging type, typical vs. atypical site, results of blood, pus, synovial fluid cultures, MRSA colonization status, Quantiferon results, PVL test positivity, treatment failure (defined as treatment escalation to broad spectrum antibiotics and/or need for surgery) and relapse at six months of follow-up. PCR tests for identification of *K. kingae* or other pathogens in case of culture-negative infections were not performed, as not included as standard of care at our facility.

### Outcomes

The following aspects of antibiotic prescription for OM and SA were evaluated:

(1) Proportion of children receiving narrow-spectrum and broad-spectrum antibiotics over different periods;
(2) Duration of therapy expressed in Days of treatment (DOT, defined as the aggregate sum of days for which any amount of a specific antimicrobial agent was administered to an individual patient) and Length of Therapy (LOT, defined as the duration of antimicrobial exposure irrespective of the number of antimicrobials administered each day) (9–11), IV DOT, DOT/LOT ratio, median DOTs of broad-spectrum antibiotics (bsDOTs), and bsDOT/DOT ratio;
(3) Length of hospital stay (LOS);
(4) Rate of patients discharged on IV integrated home care;
(5) Treatment failure, defined as the need to escalate to broad-spectrum antibiotics because of persistent fever and clinical symptoms four days after therapy start, without a decrease/with an increase of inflammatory markers, and/or the need for surgery;
(6) rate of relapse after six months.

### Data collection and sample size calculation

Clinical, demographic, diagnostic, and prescription data were manually collected from electronic medical records and stored in a password-protected data collection sheet. Privacy was guaranteed by assigning to each patient a unique, anonymized study code, and not collecting personally identifying data.

The investigations were carried out following the rules of the Declaration of Helsinki of 1975 (https://www.wma.net/what-we-do/medical-ethics/declaration-of-helsinki/), revised in 2013. The ELECTRIC study (ostEomyeLitis and sEptiC arThritis tReatment In Children) was approved by the Ethical Committee of Padua University Hospital (n.0065692). Due to the nature of the study (observational retrospective), no informed consent was required from the patients' parents or legal guardians.

### Data analysis

The variables were collected into a Microsoft® Excel database. Categorical variables were reported as number and frequency (%), while continuous variables were reported as their median and interquartile range (IQR). For the comparison of the variables among the three groups, Pearson's chi-squared tests were used for categorical variables. One-way ANOVA tests were used for continuous variables. IBM® SPSS Inc. Version 26.0 provided the results of the statistical analysis. *p*-value ≤0.05 was considered statistically significant.

## Results

**Figure 1:**
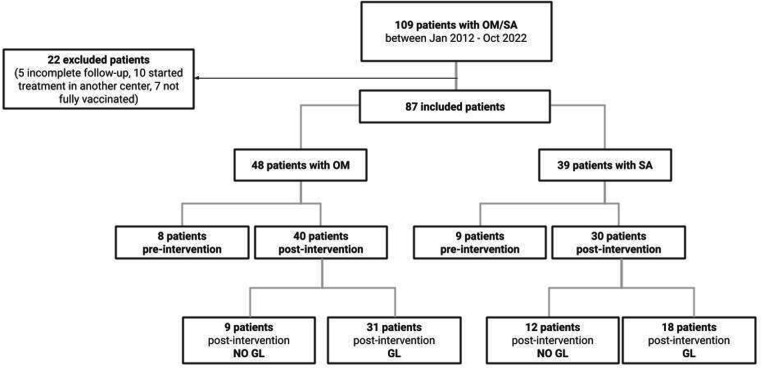
Study flow-chart. OM, osteomyelitis; SA, septic arthritis, GL, guidelines.

The total number of patients with a diagnosis of OM or SA in the study period was 109. Twenty-two patients were excluded: five for incomplete follow-up data, ten for therapy started in another center and concomitant septic status and seven for incomplete vaccination status over the post-intervention period. Of 87 included patients, 48 were diagnosed with OM (8 pre-intervention, 9 post-intervention not following GL and 31 post-intervention following GL) and 39 with SA (9 pre-intervention, 12 post-intervention not following GL and 18 post-intervention following GL), as shown in Figure 1.

### Adherence

**Figure 2:**
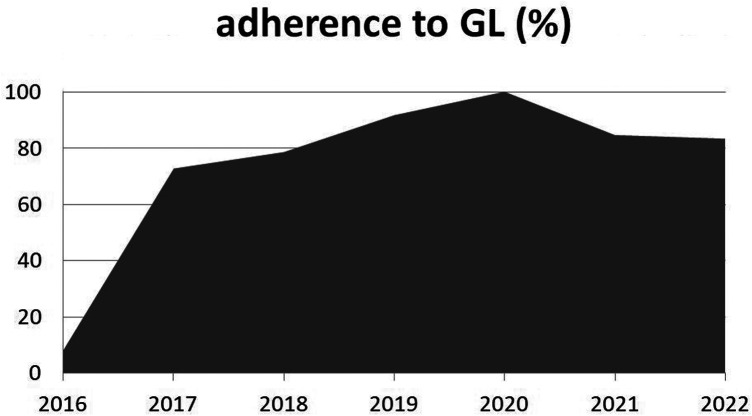
Adherence to the guidelines (GL, %) over the post-intervention years. Legend: GL, guidelines.

The overall trend of adherence to the guidelines after 2016 was constantly above 70% (Figure 2). The cases of OM/SA were highest in 2018 and 2021 (14 and 13 cases, respectively). Adherence increased abruptly from 2016 to 2017, from 8.3% to 72.7%, with a subsequent progressive raise. Maximum levels of adherence were registered in 2019 (91.6%) and 2020 (100%); since then, there has been a slight decrease to 84%. Overall adherence was 77.5% for patients with OM and 60% for SA.

### Children with OM

The baseline characteristics of the 48 children with OM are described in Table 1. The three groups were overall homogeneous, considering age, gender and body weight (*p* = 0.06, 0.95 and 0.05, respectively). There were no statistically significant differences regarding the presence of fever at onset, leukocyte and neutrophil count, and inflammation indexes (CRP, ESR, PCT). Also comparing only no GL vs. GL, the groups resulted homogeneous. None of the patients presented concomitant sepsis or multifocal disease.

**Table 1:** Characteristics of patients with OM at presentation.

		Pre-int Group (*n = *8)	Post-int no GL Group (*n = *9)	Post-int GL Group (*n = *31)	*p*-value	*p*-value (no GL vs. GL)
**Age** (median, IQR)		9.5 (8–10) years	3 (3–5) years	7 (3–10) years	0.061394 0.957901	0.148678 0.769297
**Gender** (*n*, %)	Male	5 (62.5)	6 (66.6)	19 (61.2)		
	Female	3 (37.5)	3 (33.4)	12 (38.8)		
**Body weight** (median, IQR)		27 (21–35) kg	15.5 (12.5–20) kg	25 (16.6–32.3) kg	0.051812	0.168885
**Presence of fever** (*n*, %)		6 (75)	4 (44.4)	21 (67.7)	0.348097	0.203749
**WBC count** (median, IQR)		9,860 (7,620–14,520) cells/ml	9,075 (8,722–13,590) cells/ml	8,410 (6,860–10,935) cells/ml	0.292138	0.231218
**N count** (median, IQR)		5,970 (5,472–9,085) cells/ml	4,445 (3,202–4,645) cells/ml	4,355 (3,610–5,150) cells/ml	0.072538	0.717905
**CRP-value** (median, IQR)		45 (10.4–95.7) mg/L	20.5 (7.8–49) mg/L	23 (6.7–57.2) mg/L	0.410928	0.567904
**ESR-value** (median, IQR)		29 (25–30) mm/h	29 (13–52.5) mm/h	23.5 (10–44.7) mm/h	0.875346	0.949065
**PCT-value** (median, IQR)		0.2 (0.2–0.22) ug/l	0.1 (0.1–0.17) ug/l	0.28 (0.1–0.58) ug/l	0.233273	0.160876

int, intervention; GL, guidelines; IQR, interquartile range; WBC, white blood cells; N, neutrophils; CRP, C-reactive protein; ESR, erythrosedimentation rate; PCT, procalcitonin.

Considering the performed diagnostic tests, there were no statistically significant differences among the three groups overall and no GL vs. GL, as detailed in Table 2. Most patients underwent a diagnostic MRI study, with 6 patients (75%) from the pre-intervention group, and 100% in the post-intervention groups. There were no statistically significant differences considering typical vs. atypical infection sites (*p* = 0.68). Blood culture was the most frequently performed laboratory test (100% of cases over all groups), however with a low rate of positivity, ranging from 12.5 to 22.2% and showing no significant differences among the groups (*p* = 0.86). The most frequently isolated pathogen was MSSA, with seven cases in total: one in the pre-intervention group, two in the post-intervention no GL and four in the GL group. Moreover, one case of Methicillin-sensitive *S. hominis* positivity was found in the GL group. As regards pus cultures, performed in 8 subjects overall, two were positive for MSSA, all in the pre-intervention group. One sample from the GL group showed MRSA growth and required targeted appropriate treatment.

**Table 2:** Diagnostic tests - OM.

		Pre-int Group (*n* = 8)	Post-int no GL Group (*n* = 9)	Post-int GL Group (*n* = 31)	*p*-value	*p*-value (no GL vs. GL)
**Imaging study**	**MRI scan** (*n*, %)	6 (75)	9 (100)	31 (100)	0.165587	1
	**Tc ^99^ BS** (*n*, %)	1 (12.5)	0 (0)	0 (0)		
	**CT scan** (*n*, %)	1 (12.5)	0 (0)	0 (0)		
**Site of infection** (*n*, %)	Typical	8 (100)	9 (100)	26 (83.9)	0.681911	0.475289
	High-risk	0 (0)	0 (0)	5 (16.1)		
**Blood culture** (*n*, %)	Performed	8 (100)	9 (100)	31 (100)	1	1
	Positive	1 (12.5)	2 (22.2)	5 (16.1)	0.857936	0.671918
**Pus culture** (*n*, %)	Performed	3 (37.5)	1 (11.1)	4 (12.9)	0.221338	0.886201
	Positive	2 (66.6)	0 (0)	1 (25)	0.852611	0.880303
**MRSA colonization** (*n*, %)	Screened	4 (50)	3 (33.3)	7 (22.5)	0.300118	0.511936
	Positive	0 (0)	0 (0)	1 (14.2)	0.984677	0.92265
**Quantiferon test** (*n*, %)	Performed	1 (12.5)	2 (22.2)	8 (25.8)	0.726002	0.826955
	Positive	0 (0)	0 (0)	0 (0)	–	–
**PVL test** (*n*, %)	Performed	0 (0)	0 (0)	0 (0)	–	–
	Positive	–	–	–	–	–

int, intervention; GL, guidelines; MRI, magnetic resonance imaging; Tc99 BS, Bone scintiscan; CT, computed tomography; MRSA, Methicillin-resistant *Staphylococcus aureus*; PVL, Panton-Valentine leukocidin.

The employed therapeutic regimens for OM are described in Table 3. All the 31 patients following GL received cefazolin as first line IV empiric treatment, compared to ceftriaxone and oxacillin, which was the most widely used combination regimen in both the pre-intervention and the post-intervention no GL groups (five patients each, 62.5%, and 55.5%, respectively). The choice of different regimens for initial broad-spectrum coverage was wider in the post-intervention no GL group. Five patients in the pre-intervention group (62.5%) were shifted to second line therapy on integrated home care, all with teicoplanin, compared to three patients (75%) from the post-intervention no GL group and three (33.3%) from the GL group. As regards the shift to oral therapy, 86.1% of children (25 patients) from the GL group were shifted to either a first- or second- generation cephalosporin (cefalexin or cefuroxime axetil). In the no GL group, most patients received cefalexin or clindamycin (25% each). Choices for the oral therapy were less homogeneous in the pre-intervention group, in which the preferred antibiotics were cefalexin, cepfodoxime proxetil, claritromycin and trimethoprim-sulphamethoxazole, with one patient each (25%). Even in the GL group, in some cases antibiotic choices for second line and oral therapy that differed from the guidelines were adopted. In one case this was a targeted therapy after isolation of MRSA, but in the remaining cases, considered as treatment failures, the choice was made for a lack of improvement of symptoms and CRP.

**Table 3:** Antibiotics used in the different groups - OM.

	Pre-int Group (*n = *8)	Post-int no GL Group (*n = *9)	Post-int GL Group (*n = *31)
**IV empiric treatment**			
**First line therapy**			
Cefazolin (*n*, %)	0 (0)	0 (0)	31 (100)
Cefuroxime + clindamycin (*n*, %)	0 (0)	1 (11.1)	0 (0)
Ceftriaxone + oxacillin (*n*, %)	5 (62.5)	5 (55.5)	0 (0)
Ceftriaxone + clindamycin (*n*, %)	1 (12.5)	1 (11.1)	0 (0)
Ceftriaxone + teicoplanin (*n*, %)	2 (25)	1 (11.1)	0 (0)
Teicoplanin (*n*, %)	0 (0)	1 (11.1)	0 (0)
**Second line therapy (*n***, %**)**	5/8 (62.5)	4/9 (44.4)	9/31 (29) step-up
Teicoplanin (*n*, %)	5 (100)	3 (75)	3 (33.3)
Cefazolin (*n*, %)	0 (0)	1 (25)	0 (0)
Ceftriaxone (*n*, %)	0 (0)	0 (0)	1 (11.1)
ceftriaxone + clindamycin (*n*, %)	0 (0)	0 (0)	2 (22.2)
ceftriaxone + teicoplanin (*n*, %)	0 (0)	0 (0)	2 (22.2)
teicoplanin + piperacillin/tazobactam (*n*, %)	0 (0)	0 (0)	1 (11.1)
**Oral treatment (*n***, %**)**	4/8 (50)	8/9 (88.8)	29/31 (93.5)
Cefalexin (*n*, %)	1 (25)	2 (25)	19 (65.5)
* *cefuroxime axetil (*n*, %)	0 (0)	0 (0)	6 (20.6)
cefpodoxime proxetil (*n*, %)	1 (25)	1 (12.5)	0 (0)
Cefixime (*n*, %)	0 (0)	1 (12.5)	0 (0)
amoxicillin-clavulanic acid (*n*, %)	0 (0)	1 (12.5)	0 (0)
trimethoprim/sulfamethoxazole (*n*, %)	1 (25)	1 (12.5)	2 (6.9)
Claritromycin (*n*, %)	1 (25)	0 (0)	0 (0)
Rifampin (*n*, %)	0 (0)	0 (0)	1 (3.5)
Clindamycin (*n*, %)	0 (0)	2 (25)	1 (3.5)

int, intervention; GL, guidelines; IV, intravenous.

Considering the study outcomes, the IV DOT, DOT/LOT ratio and bsDOT were significantly lower in the GL group, as shown in Table 4. The LOS was comparable among the three groups, with significantly lower rate of patients being discharged on integrated home care in the GL group (25.8%, compared to 87.5% in the pre-intervention group and 55.5% in the no GL group; *p* ≤ 0.05). The LOT, DOT and bsDOT/DOT were not significantly different over the groups.

**Table 4:** Outcomes - OM.

	Pre-int Group (*n* = 8)	Post-int no GL Group (*n = *9)	Post-int GL Group (*n = *31)	*p*-value	*p*-value (no GL vs. GL)
**LOS** (median, IQR)	12.5 (6.7–14.2) days	14 (6–16) days	13 (9.5–15) days	0.591077	0.744516
**Discharged on IV treatment** (*n*, %)	7 (87.5)	5 (55.5)	8 (25.8)	0.004434*	0.093452
**IV DOT** (median, IQR)	37.5 (28.7–44.7) days	28 (21–33) days	14 (10.5–19) days	0.031708*	0.150946
**LOT** (median, IQR)	28.5 (27–39.7) days	42 (28–47) days	34 (28–42) days	0.418227	0.353538
**DOT** (median, IQR)	45 (37.5–56) days	49 (42–61) days	34 (28–42) days	0.210495	0.113776
**DOT/LOT ratio** (median, IQR)	1.37 (1.25–1.75)	1.29 (1.16–1.47)	1 (1–1)	0.013048*	0.005618*
**bsDOT** (median, IQR)	37.5 (30.7–44.7) days	28 (21–42) days	0 (0–10.5) days	0.001375*	0.013308*
**bsDOT/DOT ratio** (median, IQR)	1 (0.78–1)	0.7 (0.38–1)	0 (0–0.26)	0.75887	0.51335
**Treatment failure** (*n*, %)	3 (37.5)	2 (22.2)	9 (29)	0.786918	0.687098
**Surgery** (*n*, %)	3 (37.5)	0 (0)	2 (6.4)	0.045767*	0.200825
**Relapse at 6 months** (*n*, %)	0 (0)	0 (0)	0 (0)	–	–

* marks statistical significance.

int, intervention; GL, guidelines; IQR, interquartile range; DOT, duration of therapy; LOT, length of therapy; bsDOT, broad spectrum DOT.

No statistically significant difference (*p* = 0.16) was observed for treatment failure, as it accounted for nine cases (29%) in the GL group, two cases (22.2%) in the post-intervention no GL group and three cases (37.5%) in the pre-intervention group. The rate of patients requiring surgery was significantly higher in the pre-intervention group (three patients, 37.5%), as compared to the no GL group, with no cases, and the GL group, with two cases (6.4%), (*p* = 0.04). There were no cases of relapse at six months.

After comparison of no GL vs. GL patients, DOT/LOT ratio and bsDOT resulted significantly inferior in the GL group (*p* = 0.005 and 0.01, respectively).

### Children with SA

As shown in Table 5, also for the 39 patients with SA, the three groups were homogeneous, with no statistically significant differences regarding baseline features, including fever at onset, WBC and neutrophil count and acute phase reactants. There were no statistically significant differences at baseline comparing the no GL and GL groups. There were no cases of sepsis or multifocal disease.

**Table 5:** Characteristics of patients with SA at presentation.

		Pre-int Group (*n* = 9)	Post-int no GL Group (*n* = 12)	Post-int GL Group (*n* = 18)	*p*-value	*p*-value (no GL vs. GL)
**Age** (median, IQR)		2 (1.5–8) years	8 (3.5–12.2) years	5.25 (2.25–8.75) years	0.190539	0.26795
**Gender** (*n*, %)	Male	5 (55.5)	4 (33.4)	9 (50)	0.543107	0.366799
	Female	4 (44.5)	8 (66.6)	9 (50)		
**Body weight** (median, IQR)		12 (10.2–27.2) kg	20 (14.5–43.7) kg	18.7 (11.4–29.5) kg	0.48906	0.231513
**Presence of fever** (*n*, %)		9 (100)	11 (91.6)	14 (77.7)	0.496995	0.957035
**WBC count** (median, IQR)		114,200 (10,150–14,280) cells/ml	12,835 (8,562–17,267) cells/ml	12,205 (8,890–15,967) cells/ml	0.590968	0.622717
**N count** (median, IQR)		5,990 (4,947–8,857) cells/ml	8,465 (4,880–11,837) cells/ml	7,350 (6,100–9,290) cells/ml	0.356069	0.439213
**CRP-value** (median, IQR)		42 (36–60) mg/l	34.2 (21.3–124.5) mg/l	45 (14–80) mg/L	0.789044	0.524246
**ESR-value** (median, IQR)		40 (24–73) mm/h	73 (56–79) mm/h	32 (19–65) mm/h	0.234865	0.088209
**PCT-value** (median, IQR)		0.8 (0.2–1.1) ug/l	0.38 (0.1–2.1) ug/l	0.1 (0.1–0.13) ug/l	0.196213	0.212431

int, intervention; GL, guidelines; IQR, interquartile range; WBC, white blood cells; N, neutrophils; CRP, C-reactive protein; ESR, erythrosedimentation rate; PCT, procalcitonin.

The analysis of diagnostic tests is displayed in Table 6. There were no statistically significant differences among the groups. Around 60% of patients in each group underwent an MRI, with a considerably superior number of children with hip involvement in the no GL group (41.7%) as compared to the GL group (5.6%), though not significantly (*p* = 0.050399). Again, blood cultures were performed in 100% of children, with no significant differences in positivity rates, ranging from 11.1% to 41.7% in the post-intervention GL and no GL groups, respectively (*p* = 0.21). The identified pathogens in the no GL group were MSSA (two cases), *S. pyogenes*, multisensitive group B *Salmonella* and multisensitive *Rothia dentocariosa* (one case each). Blood cultures in the GL group showed the growth of Methicillin-resistant *S. epidermidis* in both patients. A synovial fluid culture was performed in 22.2 to 41.7% of patients. Multisensitive *S. pneumoniae*, group B *Salmonella* and MSSA (one case each) were identified in the no GL group, while one case of MRSA positivity was found in the GL group.

**Table 6:** Diagnostic tests - SA.

		Pre-int Group (*n* = 9)	Post-int no GL Group (*n* = 12)	Post-int GL Group (*n* = 18)	*p*-value	*p*-value (no GL vs. GL)
**Imaging study**	**US** (*n*, %)	3 (33.4)	4 (33.3)	7 (38.9)	0.937067	0.938356
	**MRI scan** (*n*, %)	6 (66.6)	8 (66.7)	11 (61.1)		
**Site of infection** (*n*, %)	Typical	7 (77.7)	7 (58.3)	17 (94.4)	0.055598	0.050399
	High-risk	2 (22.3)	5 (41.7)	1 (5.6)		
**Blood culture** (*n*, %)	Performed	9 (100)	12 (100)	18 (100)	1	1
	Positive	0 (0)	5 (41.7)	2 (11.1)	0.219085	0.134152
**Synovial fluid culture** (*n*, %)	Performed	2 (22.2)	5 (41.7)	5 (27.7)	0.590598	0.429195
	Positive	0 (0)	3 (60)	1 (20)	0.753627	0.518605
**MRSA colonization** (*n*, %)	Screened	1 (11.1)	4 (33.3)	3 (16.6)	0.394379	0.537371
	Positive	0 (0)	0 (0)	1 (33.3)	0.680451	0.745024
**Quantiferon test** (*n*, %)	Performed	1 (11.1)	1 (8.3)	1 (5.5)	0.873352	0.654001
	Positive	0 (0)	0 (0)	0 (0)	–	–
**PVL test** (*n*, %)	Performed	0 (0)	0 (0)	0 (0)	–	–
	Positive	–	–	–	–	–

int, intervention; GL, guidelines; US, ultrasound; MRI, magnetic resonance imaging; MRSA, Methicillin-resistant Staphylococcus aureus; PVL, Panton-Valentine leukocidin.

Therapeutic choices in SA are summarized in Table 7: all pre-intervention patients received ceftriaxone and oxacillin in combination, while 100% of children following GL were treated with cefazolin. The choice in the no GL group fell on ceftriaxone and oxacillin or ceftriaxone and clindamycin, (41.6% and 33.3%, respectively). Seven children (77.7%) of the pre-intervention group were shifted to IV teicoplanin as second line therapy for integrated home care. In the post-intervention no GL group, two patients (16.6%) were shifted to either oxacillin, or oxacillin and rifampin for de-escalation. In the GL group, three patients (16.6%) with isolation of Methicillin-resistant *S. epidermidis* and MRSA required targeted treatment with either teicoplanin or ceftriaxone and teicoplanin.

**Table 7:** Antibiotics used in the different groups - SA.

	Pre-int Group (*n = *9)	Post-int no GL Group (*n = *12)	Post-int GL Group (*n = *18)
**IV empiric treatment**			
**First line therapy**			
Cefazolin (*n*, %)	0 (0)	0 (0)	18 (100)
Ceftriaxone + oxacillin (*n*, %)	9 (100)	5 (41.6)	0 (0)
Ceftriaxone + clindamycin (*n*, %)	0 (0)	4 (33.3)	0 (0)
Ceftriaxone + vancomycin (*n*, %)	0 (0)	1 (8.3)	0 (0)
Ceftriaxone + ampicillin (*n*, %)	0 (0)	1 (8.3)	0 (0)
Ceftriaxone (*n*, %)	0 (0)	1 (8.3)	0 (0)
**Second line therapy (*n***, %**)**	7/9 (77.7)	2/12 (16.6) step-down	3/18 (16.6) step-up
Teicoplanin (*n*, %)	7 (100)	0 (0)	1 (33.3)
Cefazolin (*n*, %)	0 (0)	0 (0)	0 (0)
Oxacillin (*n*, %)	0 (0)	1 (50)	0 (0)
Ceftriaxone + teicoplanin (*n*, %)	0 (0)	0 (0)	2 (66.7)
Oxacillin + rifampin (*n*, %)	0 (0)	1 (50)	0 (0)
**Oral treatment (*n***, %**)**	8/9 (88.8)	12/12 (100)	18/18 (100)
Cefalexin (*n*, %)	0 (0)	2 (16.6)	6 (33.3)
Cefuroxime axetil (*n*, %)	3 (37.5)	5 (41.6)	6 (33.3)
Cefpodoxime proxetil (*n*, %)	2 (25)	0 (0)	2 (11.1)
Cefixime (*n*, %)	0 (0)	2 (16.6)	1 (5.7)
Amoxicillin-clavulanic acid (*n*, %)	3 (37.5)	1 (8.4)	3 (16.6)
Rifampin (*n*, %)	0 (0)	1 (8.4)	0 (0)
Clindamycin (*n*, %)	0 (0)	1 (8.4)	0 (0)

int, intervention; GL, guidelines; IV, intravenous.

The most widely used molecules for oral therapy were cefalexin and cefuroxime axetil in the post-intervention groups, while cefpodoxime proxetil and amoxicillin-clavulanate were mostly prescribed over the pre-intervention years.

Results for outcome variables are detailed in Table 8. A statistically significant difference among the groups emerged for LOS, IV DOT, DOT/LOT ratio and bsDOT, as well as for the proportion of patients discharged on IV therapy. IV DOT, DOT/LOT ratio and bsDOT were lowest in the post-intervention GL group (*p* = 0.02, ≤0.05 and ≤0.05, respectively), showing a lower use of combination and broad-spectrum regimens. LOS was significantly lower (*p* ≤ 0.05) in both the pre-intervention group and GL group, with no difference between the two groups (*p* = 0.94). The rate of children discharged with integrated home care was lower in the post-intervention groups (*p* ≤ 0.05). LOT, DOT and bsDOT/DOT were lowest in the GL group, but not significantly. No statistically significant differences were found for treatment failure, including surgery. There was no relapse at six months.

**Table 8:** Outcomes - SA.

	Pre-int Group (*n = *9)	Post-int no GL Group (*n = *12)	Post-int GL Group (*n = *18)	*p*-value	*p*-value (no GL vs. GL)
**LOS** (median, IQR)	7 (6–12) days	13 (10.5–15.7) days	8 (6.2–9.7) days	0.00486*	0.005154*
**Discharged on IV treatment** (*n*, %)	7 (77.7)	1 (8.3)	4 (22.2)	0.00167*	0.317311
**IV DOT** (median, IQR)	28 (26–33)	22.5 (18–28.5)	8 (7–14)	0.021583*	0.018736*
**LOT** (median, IQR)	28 (17–29) days	28 (21–30) days	21.5 (14.2–27.7) days	0.184855	0.103866
**DOT** (median, IQR)	42 (29–44) days	38 (31.2–44.7) days	21.5 (14.2–27.7) days	0.054293	0.030863*
**DOT/LOT ratio** (median, IQR)	1.51 (1.46–1.60)	1.44 (1.33–1.62)	1 (1–1)	0.001869*	0.001428*
**bsDOT** (median, IQR)	33 (28–42) days	23.5 (21–27.5) days	0 (0–9.5) days	0.000396*	0.001352*
**bsDOT/DOT ratio** (median, IQR)	0.86 (0.73–1)	0.63 (0.56–0.70)	0 (0–0.43)	0.081606	0.02209*
**Treatment failure** (*n*, %)	1 (11.1)	1 (8.3)	3 (16.6)	0.787438	0.912701
**Surgery** (*n*, %)	1 (11.1)	1 (8.3)	1 (5.5)	0.873352	0.654001
**Relapse at 6 months** (*n*, %)	0 (0)	0 (0)	0 (0)	–	

* marks statistical significance.

int, intervention; GL, guidelines; IQR, interquartile range; DOT, duration of therapy; LOT, length of therapy; bsDOT, broad spectrum DOT.

After comparison of no GL and GL groups, LOS, IV DOT, DOT, DOT/LOT ratio, bsDOT and bsDOT/DOT ratio resulted significantly inferior in the GL group (Table 8).

## Discussion

In this monocentric, retrospective, observational study, the implementation of guidelines for the treatment of uncomplicated pediatric OM and SA proved its efficacy in decreasing the extensive use of broad-spectrum antibiotics both for the IV empiric treatment and for the subsequent oral therapy shift. In fact, we observed lower bsDOTs in the post-intervention GL groups for both OM and SA, and a significantly lower IV DOTs and DOT/LOT ratios, indicating a lower use of combination therapy. Remarkably, these findings emerged even more strongly comparing children with SA from no GL vs. GL groups, which had more similar numerosity. As compared to the no GL group, children with SA that received a narrow-spectrum treatment following the guidelines had a significantly shorter hospital stay and were exposed to a significantly inferior use of combination therapy, intravenously and overall, as well as broad-spectrum antibiotics. Comparing the three groups, however, LOT, DOT and bsDOT/DOT were generally lower in the post-intervention groups following the guidelines, as the small sample size may have played a role in limiting the statistical significance of the findings. The tendency of an inferior LOS in the pre-intervention period may be explained by the fact that almost all patients were discharged on IV teicoplanin with integrated home care. There was a significant decrease in this practice over the post-intervention years, with a significantly lower proportion of patients with OM discharged on IV therapy in the GL group. This procedure while ensuring a more cautious approach, may expose children to complications related to peripherally inserted intravenous catheters (PICC), requiring more frequent post-discharge visits and sometimes hospitalization therefore implying high healthcare costs. These considerations have been discussed and underlined in the retrospective study by Keren et al., that therefore suggested to discharge patients on equally effective oral alternatives (12).

In our study, the rate of treatment failure in children treated with the narrow spectrum regimen was not different from those with broad spectrum approaches and, furthermore, no relapse cases were observed at follow-up. Interestingly, among patients with OM, there was a significant drop in patients requiring surgery in the post-intervention period, also considering an increasing number of cases of BJI. In our opinion, this result suggests that the treatment proposed by our guidelines did not lead to a higher risk of complications, was safe and therefore non-inferior to the previous broad spectrum regimen.

Possible reasons behind treatment failure may be an involvement of the hips, which are known to be associated with worse outcomes, resistant pathogens, such as MRSA, or unusual bacteria (e.g., *Salmonella*) as causative agents. Moreover, another plausible hypothesis may be Panton-Valentine leukocidin (PVL) production, which remains unknown in our cohort, due to a lack of testing. According to the literature, the evolution to severe musculoskeletal infections, though rare, is related to PVL, which is known to determine a more severe local disease and increased systemic inflammatory response (13–15). Moreover, *K. kingae* may present a decreased susceptibility to oxacillin, so this treatment should not be used on an empiric basis in contexts where its incidence is high (16). The treatment of severe invasive musculoskeletal infection, as stated above, usually needs drainage of the abscesses and combination with a toxin inhibitor antibiotic, like clindamycin, rifampin, or linezolid. The retrospective nature of our study does not allow us to discern with certainty whether all the cases that were identified as treatment failure needed step-up treatment, or if the escalation was decided on a cautious approach.

International guidelines for pediatric bone and joint infection suggest the initial use of IV cefazolin or oxacillin (17–21), which are considered therapeutically equivalent, based on *in vitro* data and retrospective studies, for empiric IV treatment with coverage of MSSA (7, 8). The age of the patient and local, recent resistance patterns must always be considered in the choice of an empiric treatment. The total duration of antibiotic therapy in acute non-complicated OM was historically four to six weeks, with more recent studies and guidelines now suggesting three-four weeks, while two-three weeks are recommended for SA (22, 23). Shift to oral therapy is considered as equivalent to prolonged IV treatment, with fewer complications (12, 24). Early switch to oral antibiotics is suggested as safe in non-complicated musculoskeletal infection when the child is afebrile, symptoms are improving and CRP has decreased of 50% from maximum value (7, 18, 25).

All children with a suspicion of BJI should undergo a baseline assessment with complete blood count and CRP. The main laboratory test to monitor response to antibiotic treatment is CRP, as ESR is a poorer predictor, because it tends to remain elevated for several weeks after resolution of the acute phase of the infection. A blood culture should always be obtained before starting antibiotic treatment, despite the low positivity rates (10%–40%), as emerged also from our study, in line with data from the literature (7). As regards imaging, MRI is the gold standard for diagnosis of OM, while US is very sensitive in identifying joint effusion in septic arthritis. In our study, an MRI was performed in more than half of the cases of SA, after US, as it provides excellent definition of the soft tissues and may exclude or confirm concomitant OM. The need of surgical interventions in children with OM lacks consensus as for need, timing and extent, but is to be considered in case of a scarce response to antibiotics, with persistent fever, symptoms and/or elevated CRP, presence of abscesses or suspicion of other complications. In case of SA, a joint drainage with arthrocentesis is recommended after the diagnosis, while surgical drainage through arthrotomy is usually reserved in case of treatment failure. In our cohort arthrocentesis was not universally employed.

As regards adherence, a positive trend was maintained over the post-intervention years, with an overall rate above at least 70% since late 2016, peaking in 2020, when it reached 100%, after *ad hoc* training sessions had been offered to the staff. The drop, however small, that was registered soon after 2020 may be explained by the whole in-hospital situation due to the COVID-19 pandemic, in which continuing education lectures were difficult to offer. Even though data for 2022 are not complete, the most recent trends underline the need for continuous implementation of guidelines, to achieve optimal compliance levels over time.

Considering the slightly lower adherence registered for SA children, patient factors might have had a role. Notably, although no significant factors emerged from our analysis, younger age and especially the involvement of a high-risk infection site like the hips may have been determinants for choosing a more cautious approach. To some degree, other possible reasons for a lack of adherence might rely on a tendency to remain attached to long-standing previous practice, especially at a distance to implementation/reminder lectures. As reported in a recent review by DeRonde et al., the implementation of antibiotic stewardship programs may have a major role in the improvement of the care of children with OM or SA. In fact, offering easily accessible pathways for clinical practice, antibiotic stewardship can help to target and optimize treatment, to guarantee the narrowest effective therapy and, at the same time, to limit the selection pressure and the development of antimicrobial resistance (26).

### Limitations

This study presents some limitation, first, due to its retrospective nature and single-centre setting. Despite quite a long observation time of almost 11 years, another considerable limitation is the small sample size, partly explained by the rarity of the considered diseases. Nevertheless, this might have contributed, to some degree, to reducing the power of the study. Last, although there has been a considerable improvement in antibiotic prescriptions, the absolute volume of IV antibiotics being prescribed in our cohort, including children in the GL group and especially with OM, still seems excessive. Future directions indeed lead to the prescription of the shortest possible IV regimen, even towards the exclusive use of oral therapy for BJI, following the results of the OVIVA trial, conducted on adults, which demonstrated the non-inferiority of oral antibiotics during the first six weeks for complex orthopedic infection, as assessed by treatment failure at one year (27).

## Conclusions

Our results, in line with the most recent ESPID clinical practice guidelines for OM and SA, showed the applicability, efficacy, and safety of a narrow-spectrum empirical antibiotic regimen with a first-generation cephalosporin, followed by oral monotherapy with first/second-generation cephalosporins, which was non-inferior to broad spectrum, combination regimens for the treatment of uncomplicated pediatric BJI.

## Data Availability

The raw data supporting the conclusions of this article will be made available by the authors, without undue reservation.
